# PI3K/mTOR Signaling Pathway Dual Inhibition for the Management of Neuroinflammation: Novel Insights from In Vitro Models

**DOI:** 10.3390/biom15050677

**Published:** 2025-05-07

**Authors:** Alessio Ardizzone, Sarah Adriana Scuderi, Giovanna Casili, Rossella Basilotta, Emanuela Esposito, Marika Lanza

**Affiliations:** Department of Chemical, Biological, Pharmaceutical and Environmental Sciences, University of Messina, 98166 Messina, Italy; aleardizzone@unime.it (A.A.); sascuderi@unime.it (S.A.S.); gcasili@unime.it (G.C.); rossella.basilotta@unime.it (R.B.); mlanza@unime.it (M.L.)

**Keywords:** neuroinflammation, PI3K/mTOR pathway, in vitro models, neurodegeneration

## Abstract

Neuroinflammatory responses are central to the pathogenesis of neurodegenerative diseases, affecting cells of both neuronal and glial origin that respond to immune-driven inflammatory stimuli. The PI3K/mTOR signaling pathway is essential for the regulation of these neuroinflammatory processes and is therefore a promising target for therapeutic intervention. Here, we investigated the consequences of PI3K/mTOR pathway inhibition on neuroinflammation employing PF-04691502, an agent with combined PI3K and mTOR inhibitory activity. We treated SH-SY5Y, C6, BV-2, and Mo3.13 cell lines with PF-04691502 at concentrations of 0.1, 0.5, and 1 µM to assess the modulation of neuroinflammatory responses. To induce inflammation, cells were stimulated with lipopolysaccharide (LPS, 1 μg/mL) and interferon-gamma (IFN-γ, 100 U/mL). The results from the MTT assays demonstrated that PI3K/mTOR inhibition preserved cell viability at 0.5 and 1 µM across all of the cell lines, indicating its potential to mitigate inflammation-driven cytotoxicity. Subsequent ELISA assays revealed a marked decrease in the NF-κB and pro-inflammatory cytokine levels, confirming the effective suppression of inflammation through PI3K/mTOR inhibition. In addition, the SH-SY5Y cell line was exposed to MPP+ to simulate Parkinson’s disease (PD)-like toxicity; then, cell viability, PD-associated markers, and apoptotic indicators were assessed. Our results indicate that inhibition of the PI3K/mTOR signaling axis may alleviate neurodegenerative processes by modulating both neuroinflammatory responses and apoptotic pathways. These findings highlight the therapeutic promise of targeting PI3K/mTOR in the context of neurodegenerative disorders and support the need for further validation through in vivo and clinical investigations.

## 1. Introduction

Neuroinflammatory processes are a key feature underlying several neurodegenerative disorders, including Alzheimer’s (AD) and Parkinson’s disease (PD), both marked by progressive neuronal loss, cognitive impairments, and motor abnormalities [1]. Neuroinflammation is largely mediated by activated glial cells, especially microglia and astrocytes, which are essential for maintaining central nervous system (CNS) homeostasis [2]. Activated microglia and astrocytes contribute to the neuroinflammatory milieu by producing cytokines such as tumor necrosis factor-α (TNF-α), interleukin-1β (IL-1β), and interleukin-6 (IL-6), which drive the initiation and maintenance of inflammation [2,3]. Although acute neuroinflammation contributes to host defense and tissue healing, its chronic activation can result in neuronal injury, impaired synaptic function, and the advancement of neurodegenerative processes [1,3].

The phosphoinositide 3-kinase (PI3K) and mechanistic target of rapamycin (mTOR) signaling pathways are central regulators of cellular survival, metabolism, and immune response [4,5]. PI3K activation triggers downstream phosphorylation events through protein kinase B (AKT), which, in turn, activates mTOR complex 1 (mTORC1) [6,7].

This signaling cascade controls microglial activation, cytokine production, and oxidative stress responses [8,9]. However, dysregulated PI3K/mTOR signaling contributes to neuroinflammation by increasing reactive oxygen species (ROS) release, promoting inflammatory gene expression via nuclear factor kappa B (NF-κB), and impairing autophagic clearance of toxic protein aggregates like amyloid-beta (Aβ) and alpha-synuclein (a-syn) [10,11].

Given the interplay between PI3K/mTOR and neuroinflammatory pathways, the dual inhibition of these kinases has emerged as a possible therapeutic strategy [10]. In contrast to agents that selectively inhibit mTOR (such as rapamycin) or target PI3K alone, dual PI3K/mTOR inhibitors simultaneously block all catalytic isoforms of PI3K as well as both mTOR complexes (mTORC1 and mTORC2) [12,13]. This broader inhibition profile allows them to bypass the compensatory feedback mechanisms typically seen with mTORC1 inhibition alone, resulting in enhanced therapeutic efficacy [12,13].

Among them, PF-04691502 has gained great interest in its field of research [14,15]. Specifically, PF-04691502 is a highly potent and orally available small-molecule compound that selectively inhibits the PI3K and mTOR signaling pathways [16]. PF-04691502 was originally investigated for its antineoplastic properties due to its ability to suppress tumor cell growth and viability [17]. However, its potential in the context of neuroinflammation has not yet been fully investigated.

Therefore, building on these observations, the present study investigates the impact of PF-04691502 on neuroinflammatory processes linked to neurodegenerative diseases by modulating the PI3K/mTOR signaling pathway through in vitro models.

## 2. Materials and Methods

### 2.1. Materials

PF-04691502 (Cat. N°: HY-15177) was obtained from MedChemExpress (Monmouth Junction, NJ, USA). DMSO was used to prepare the stock solution of PF-04691502. All reagents and materials employed in this study were purchased from Sigma-Aldrich (Milan, Italy), unless specified otherwise. The highest commercially available grade of each compound was used throughout the experiments.

### 2.2. Cell Lines

#### 2.2.1. C6

The rat C6 glioma cell line (ATCC^®^ CCL-107^TM^, Manassas, VA, USA) was obtained from the American Type Culture Collection (Manassas, VA, USA). Cells were grown in 75 cm^2^ culture flasks containing Dulbecco’s Modified Eagle Medium (DMEM; Sigma-Aldrich, Milan, Italy), supplemented with 10% fetal bovine serum (FBS), 2 mM L-glutamine, 100 U/mL penicillin, and 100 μg/mL streptomycin (all reagents were from Sigma-Aldrich, Milan, Italy). Cultures were maintained at 37 °C in a humidified incubator with 5% CO_2_ atmosphere.

#### 2.2.2. SH-SY5Y

The human SH-SY5Y neuroblastoma cell line (ATCC^®^ CRL-2266^TM^) was obtained from the American Type Culture Collection (Manassas, VA, USA). This subclone originates from a metastatic bone tumor and can be differentiated into neuron-like cells exhibiting both morphological and biochemical features of mature neurons upon exposure to 100 nM retinoic acid (RA; Sigma-Aldrich, Milan, Italy).

Cells were cultured in a 1:1 mixture of Dulbecco’s Modified Eagle Medium (DMEM) and Ham’s F12 medium (Sigma-Aldrich, Milan, Italy), supplemented with 2 mM L-glutamine, 1 mM sodium pyruvate, 10% fetal bovine serum (FBS), and 50 μg/mL streptomycin (all reagents were from Sigma-Aldrich, Milan, Italy). Cell cultures were maintained at 37 °C in a humidified atmosphere containing 5% CO_2_.

#### 2.2.3. BV-2

The BV-2 cell line, a murine microglial cell model derived from C57BL/6 mice, was obtained from the Interlab Cell Line Collection (ICLC), Cell Bank IRCCS AOU San Martino IST (Genoa, Italy). Cells were cultured in RPMI 1640 medium (Sigma-Aldrich, Milan, Italy) supplemented with 10% fetal bovine serum (FBS), 100 U/mL penicillin, and 100 μg/mL streptomycin (Sigma-Aldrich, Milan, Italy). Cultures were maintained at 37 °C in a humidified incubator with 5% CO_2_.

#### 2.2.4. Mo3.13

The Mo3.13 cell line, a human immortalized oligodendrocyte hybrid derived from a human–human fusion, was obtained from Cedarlane Laboratories (CLU301; Burlington, ON, Canada). Cells were maintained in Dulbecco’s Modified Eagle Medium (DMEM; Sigma-Aldrich, Milan, Italy), supplemented with 10% fetal bovine serum (FBS), 100 U/mL penicillin, and 100 μg/mL streptomycin (Sigma-Aldrich, Milan, Italy). Cultures were incubated at 37 °C in a humidified atmosphere containing 5% CO_2_.

### 2.3. Experimental Procedures

A total of 2 × 10^4^ cells per well were seeded into 96-well plates (Corning Cell Culture, Corning, NY, USA). To identify effective concentrations of PF-04691502 with minimal cytotoxicity, a colorimetric assay using 3-(4,5-dimethylthiazol-2-yl)-2,5-diphenyltetrazolium bromide (MTT) was conducted across a concentration range from 0.1 to 10 μM. The tested concentrations were selected based on prior literature and preliminary dose–response evaluations previously carried out in our laboratory using the MTT assay [18].

Following the selection of higher PF-04691502 concentrations associated with minimal cytotoxicity, an in vitro inflammatory model was established using lipopolysaccharide (LPS, 1 μg/mL) and interferon-gamma (IFN-γ, 100 U/mL) [19], in the presence or absence of PF-04691502 at 0.1, 0.5, and 1 μM.

All cell lines were divided into the following experimental groups:

Control group (CTR): cells were cultured with normal culture medium.

LPS/IFNγ group: cells were exposed to LPS (1 μg/mL) and IFN-γ (100 U/mL) for 24 h.

LPS/IFNγ + PF-04691502 0.1 μM group: cells were exposed to LPS (1 μg/mL) and IFN-γ (100 U/mL) and treated with PF-04691502 0.1 μM for a 24 h period.

LPS/IFNγ + PF-04691502 0.5 μM group: cells were exposed to LPS (1 μg/mL) and IFN-γ (100 U/mL) and treated with PF-04691502 0.5 μM for a 24 h period.

LPS/IFNγ + PF-04691502 1 μM group: cells were exposed to LPS (1 μg/mL) and IFN-γ (100 U/mL) and treated with PF-04691502 1 μM for a 24 h period.

In another set of experiments, SH-SY5Y cells, after differentiation with RA (100 nM) for 24 h, were challenged with 1-Methyl-4-phenylpyridinium (MPP^+^) (Sigma-Aldrich) (1 mM) [20] for an additional 24 h, in the presence or absence of PF-04691502 (0.1, 0.5, and 1 μM), to reproduce an in vitro model of PD.

SH-SY5Y cells were divided into the following experimental groups:

CTR: cells were cultured with normal culture medium.

MPP^+^ group: cells were exposed to MPP^+^ (1 mM) for 24 h.

MPP^+^ + PF-04691502 0.1 μM group: cells were exposed to MPP^+^ (1 mM) plus PF-04691502 0.1 μM for 24 h.

MPP^+^ + PF-04691502 0.5 μM group: cells were exposed to MPP^+^ (1 mM) plus PF-04691502 0.5 μM for 24 h.

MPP^+^ + PF-04691502 1 μM group: cells were exposed to MPP^+^ (1 mM) plus PF-04691502 1 μM for 24 h.

### 2.4. MTT Assay

The survival rate of cells was evaluated through a mitochondria-dependent colorimetric assay using the tetrazolium salt MTT. After treatment, MTT was added to each well at a final concentration of 0.2 mg/mL and incubated for 1 h at 37 °C, as previously described [21]. Absorbance was recorded at 570 nm.

### 2.5. ELISA Kit

ELISAs were performed on cell lysates and the optical density (OD) was recorded at a wavelength of 450 nm, according to manufacturers’ instructions and the methodology used in our previous studies [22]. Cell lysates were prepared following the manufacturer’s instructions for each ELISA kit. In general, adherent cells were first detached using trypsin and then collected by centrifugation. Once collected, the cells were washed three times with PBS to remove any residual contaminants.

Next, the cells were resuspended in PBS and subjected to the lysis process. Then, cells were centrifuged at 1000× *g* for 15 min at 4 °C to remove cellular debris, leaving the lysate ready for analysis. Finally, lysates were stored at −80 °C for their future use.

The amount of protein was determined to ensure equal protein loading across all samples prior to performing the ELISA, in order to avoid potential confounding effects.

### 2.6. Immunofluorescence

Immunofluorescence staining was executed as previously described [23]. SH-SY5Y cells in 1 mL were plated on a microscope slide (2 × 10^5^). Following 24 h of incubation, cells were differentiated with 100 nM retinoic acid (RA) for an additional 24 h, after which they were exposed to MPP^+^ and PF-04691502. After 24 h of treatment, SH-SY5Y cells were fixed with paraformaldehyde (PFA) 4% for 30 min. Anti-Tyrosine Hydroxylase (1:100, Cat. No. bsm-52574R, Bioss, Woburn, MA, USA) and a-synuclein (1:100, sc-12767; Santa Cruz Biotechnology, Dallas, TX, USA) were added to the cells overnight in a humidified chamber. Later, secondary antibody incubation with Alexa Fluor-488 or Alexa Fluor-594 (1:1000 in PBS, *v*/*v*, Invitrogen; Waltham, MA, USA) for 3 h was performed. Sections were washed in PBS and 4′,6′-diamidino-2-phenylindole (DAPI; Hoechst, Frankfurt, Germany) (2 μg/mL) was added to the cells for nuclear staining. Images were captured at 40× magnification using a fluorescence microscope (Nikon Eclipse Ci-L, NIKON CORPORATION, Tokyo, Japan).

### 2.7. Statistical Analysis

After assessing the normal or not normal distribution of data, statistical analysis was performed using a One-Way ANOVA test, followed by multiple comparisons through Bonferroni’s test. Data are presented as the mean ± standard deviation (SD) from three independent experiments. All statistical evaluations were carried out using GraphPad Prism 9.00 software. Statistical significance was defined as *p* < 0.05.

## 3. Results

### 3.1. Evaluating the Concentration-Dependent Cytotoxic Effects of the Dual PI3K/mTOR Inhibitor PF-04691502 in Neural and Glial Models

To identify the optimal concentrations of PF-04691502 with poor toxicity, we evaluated cell survival in C6, SH-SY5Y, BV-2, and Mo3.13 cells after treatment with the compound at 0.1, 0.5, 1, 2, 5, and 10 μM.

**Figure 1:**
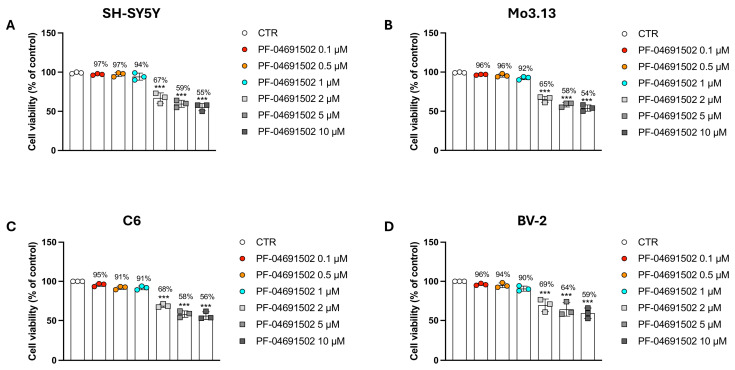
Evaluation of PF-04691502 cytotoxicity in C6, SH-SY5Y, BV-2, and Mo3.13 cells. Cell viability was assessed following treatment with PF-04691502 (0.1, 0.5, 1, 2, 5, and 10 μM) for 24 h using MTT assay (**A**–**D**). Data are presented as mean ± SD from three independent experiments. One-Way ANOVA. *** *p* < 0.001 vs. CTR.

Across all of the tested cell lines, the lowest concentrations of PF-04691502 (0.1, 0.5, and 1 μM) did not exhibit significant toxicity, maintaining good cell vitality (Figure 1A–D). In contrast, at 2, 5, and 10 µM, the treatment showed cytotoxic effects in a concentration-dependent manner, with a more than 30% reduction in cell viability (Figure 1A–D).

Based on these preliminary analyses, the concentrations of 2, 5, and 10 μM were excluded from further experiments due to their cytotoxic effects.

### 3.2. The Dual PI3K/mTOR Inhibitor PF-04691502 Modulates Inflammation in Neural and Glial Cells Induced by LPS/IFNγ

LPS and IFN-γ stimulation was used to elicit an inflammatory response and assess the effects of PF-04691502.

**Figure 2:**
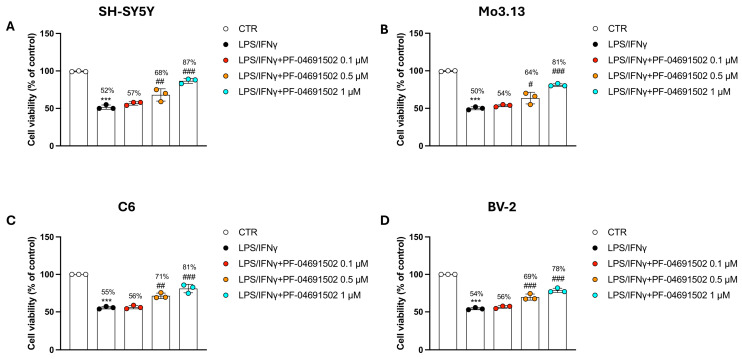
Dual Inhibition of PI3K/mTOR by PF-04691502 protects cells against LPS/IFNγ-induced inflammatory damage. Graphs related to MTT experiments following inflammatory stimuli (**A**–**D**). Data are reported as mean ± SD from three independent experiments. One-Way ANOVA. *** *p* < 0.001 vs. CTR; ^#^ *p* < 0.05 vs. LPS/IFNγ; ^##^ *p* < 0.01 vs. LPS/IFNγ; ^###^ *p* < 0.001 vs. LPS/IFNγ.

Firstly, we assessed the cytotoxicity in the neural and glial cell lines using an LPS/IFNγ-induced inflammation model. LPS/IFNγ induced a decrease in the cellular survival for each cell line investigated compared to the CTR group; however, PF-04691502 at concentrations of 0.5 and 1 μM re-established cell viability (Figure 2A–D). No significance was observed with PF-04691502 at a concentration of 0.1 μM (Figure 2A–D).

### 3.3. Dual Inhibition of PI3K/mTOR by PF-04691502 Modulates NF-κB Pathway, iNOS, and COX-2 Levels in a Concentration-Dependent Manner

To investigate the anti-inflammatory effects of PF-04691502, we analyzed the activation of the NF-κB signaling pathway and the levels of key inflammatory mediators, iNOS and COX-2, across all of the tested cell lines.

**Figure 3:**
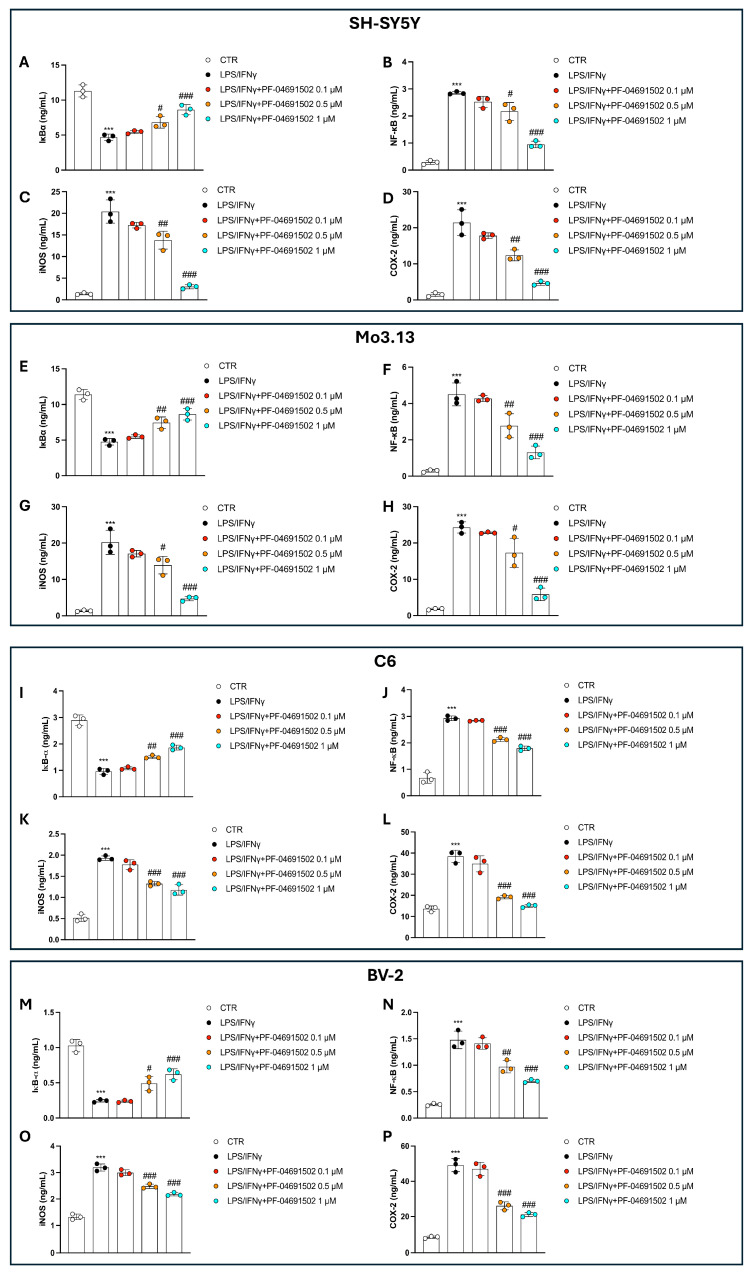
PF-04691502 reduces NF-κB activation and downregulates iNOS and COX-2 levels. ELISA kits to evaluate NF-κB, IkB-α, iNOS, and COX-2 across all cell lines (**A**–**P**). Data are indicated as mean ± SD from three independent experiments. One-Way ANOVA. *** *p* < 0.001 vs. CTR; ^#^ *p* < 0.05 vs. LPS/IFNγ; ^##^ *p* < 0.01 vs. LPS/IFNγ; ^###^ *p* < 0.001 vs. LPS/IFNγ.

Stimulation with LPS/IFNγ led to robust NF-κB pathway activation, accompanied by a marked increase in the levels of iNOS and COX-2 when compared to the CTR group (Figure 3A–P).

The treatment with PF-04691502 at 0.1 μM did not significantly alter NF-κB activation, and also did not considerably reduce the iNOS and COX-2 levels (Figure 3A–P), suggesting that this concentration is insufficient to counteract the inflammatory response.

Contrastingly, 0.5 μM and 1 μM of PF-04691502 effectively attenuated NF-κB activation and significantly downregulated the iNOS and COX-2 levels in a concentration-dependent way (Figure 3A–P). These effects were consistently observed across all cell lines, further supporting the anti-inflammatory properties of PF-04691502 at higher concentrations.

### 3.4. PF-04691502 Reduces TNF-α, IL-1β, and IL-6 Levels in a Concentration-Dependent Manner Through PI3K/mTOR Inhibition

**Figure 4:**
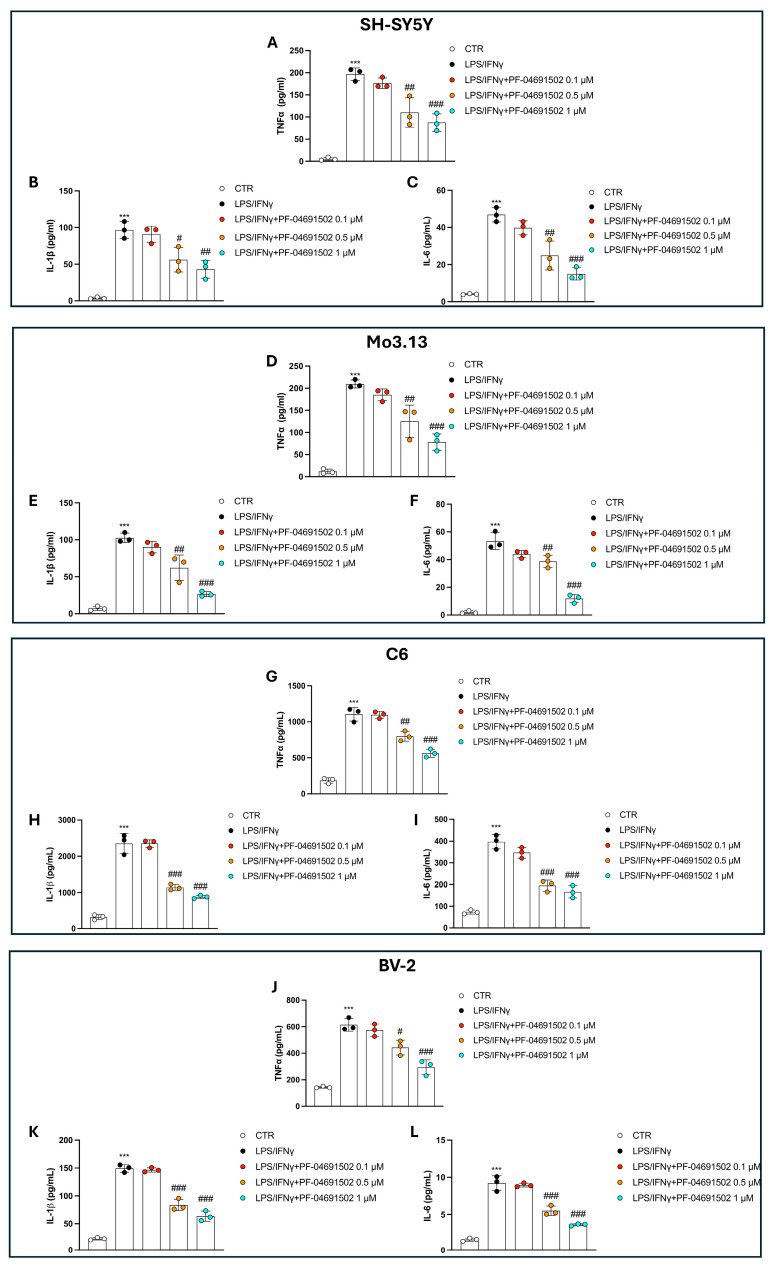
PF-04691502 modulates TNF-α, IL-1β, and IL-6 levels. Evaluation of TNF-α, IL-1β, and IL-6 by ELISA kits, across all cell lines (**A**–**L**). Data are reported as mean ± SD from three independent experiments. One-Way ANOVA. *** *p* < 0.001 vs. CTR; ^#^ *p* < 0.05 vs. LPS/IFNγ; ^##^ *p* < 0.01 vs. LPS/IFNγ; ^###^ *p* < 0.001 vs. LPS/IFNγ.

To assess the impact of PF-04691502 on pro-inflammatory cytokines, we evaluated the TNF-α, IL-1β, and IL-6 levels in cells stimulated with LPS/IFNγ by employing ELISA kits. LPS/IFNγ exposure considerably elevated the TNF-α, IL-1β, and IL-6 levels compared to those of the CTR group, confirming the successful induction of inflammation (Figure 4A–L).

At the lowest concentration of PF-04691502 (0.1 μM), no significant reduction in cytokine production was observed, indicating that this concentration was insufficient to modulate the inflammatory response (Figure 4A–L). The treatments with PF-04691502 at 0.5 μM and 1 μM resulted in a clear concentration-dependent effect, lowering the TNF-α, IL-1β, and IL-6 levels (Figure 4A–L).

### 3.5. PF-04691502 Dual Inhibition of PI3K/mTOR Protects SH-SY5Y Cells from MPP^+^-Induced Neurodegeneration

In this set of experiments, SH-SY5Y cells were exposed to MPP^+^, a well-established neurotoxin, and also to previously chosen concentrations of PF-04691502 (0.1, 0.5, and 1 μM) to assess its potential neuroprotective effects.

**Figure 5:**
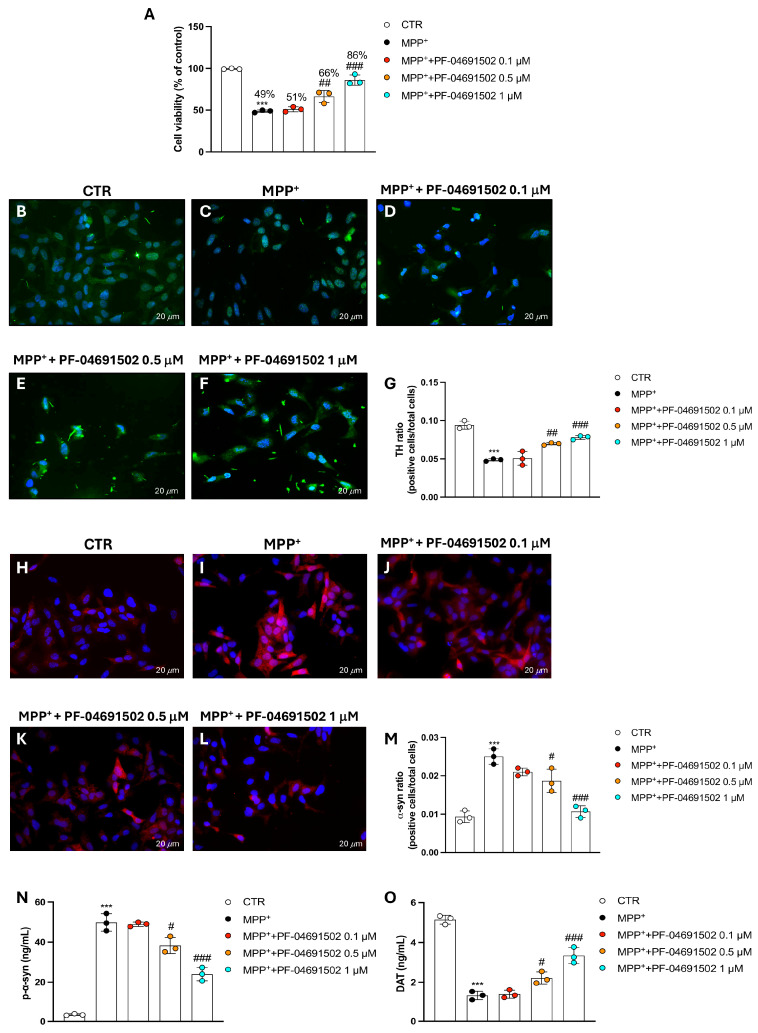
Protective effects of PF-04691502 on MPP^+^-induced neurodegeneration in SH-SY5Y cells. Graph presenting results of MTT for the assessment of cell survival (**A**). TH and α-syn were determined through immunofluorescence for all experimental groups (**B**–**F** and **H**–**L** respectively); immunofluorescence scores for both TH and α-syn are reported (**G** and **M** respectively). p-α-syn and DAT levels were measured by ELISA assay (**N**,**O**). Data are presented as mean ± SD from three independent experiments. One-Way ANOVA. *** *p* < 0.001 vs. CTR; ^#^ *p* < 0.05 vs. MPP^+^; ^##^ *p* < 0.01 vs. MPP^+^; ^###^ *p* < 0.001 vs. MPP^+^.

The MTT technique was used to detect cell survival, which revealed that the treatment with PF-04691502, only at 0.5 and 1 μM, significantly improved the cell viability compared to the MPP^+^-treated group (Figure 5A).

TH expression, a marker for dopaminergic neurons, was assessed through immunofluorescence. The data showed a considerable decrease in TH-positive cells in the MPP^+^ group (Figure 5C, score 5G) compared to in the CTR group (Figure 5B, score 5G). A marked recovery of dopaminergic neurons was instead detected in the PF-04691502-treated groups at the highest concentrations of 0.5 and 1 μM compared to the MPP^+^ group, indicating that PF-04691502 can protect dopaminergic neurons from MPP^+^-induced damage (Figure 5D–F, score 5G).

Moreover, α-syn, a protein associated with neurodegeneration, was analyzed by immunofluorescence. The MPP^+^ group exhibited increased α-syn-positive cells (Figure 5I, score 5M) compared to in the CTR group (Figure 5H, score 5M). PF-04691502 addition led to a significant reduction in α-syn-positive cells in a concentration-dependent manner (Figure 5J–L, score 5M), suggesting that PF-04691502 may modulate the accumulation of α-syn, potentially reducing toxic aggregation. The ELISA kit for p-α-syn confirmed these results (Figure 5N).

In addition, the DAT levels were evaluated using an ELISA kit. In the MPP^+^-treated cells, DAT was significantly lowered compared to in the CTR group (Figure 5O), indicating impaired dopaminergic function. However, the concentrations of PF-04691502 of 0.5 and 1 μM restored the DAT levels in the MPP^+^-treated cells, suggesting a preservation of dopaminergic function (Figure 5O). In contrast, the 0.1 μM concentration of PF-04691502 did not exhibit any statistically noteworthy effect on DAT levels when compared to the MPP^+^ group (Figure 5O).

### 3.6. Modulation of PI3K/mTOR Signaling and Apoptotic Pathways by PF-04691502 in MPP^+^-Treated SH-SY5Y Cells

After MPP^+^ stimulation, we assessed key markers involved in cell survival and apoptosis pathways, including PI3K and mTOR, which are known to be inhibited by PF-04691502.

**Figure 6:**
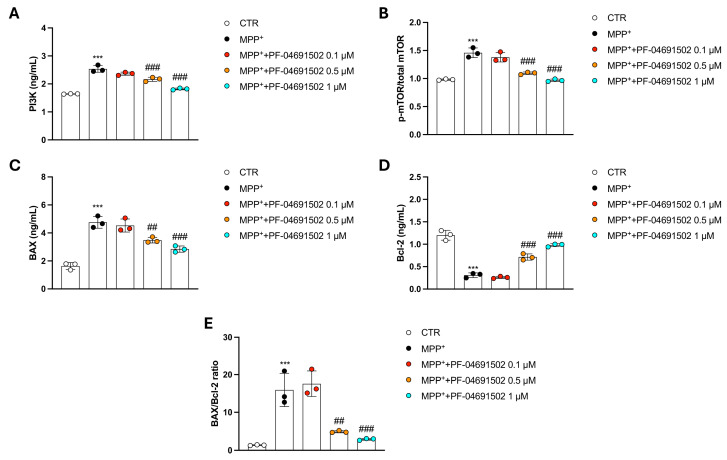
Evaluation of PI3K/mTOR signaling and apoptotic markers in MPP^+^-treated SH-SY5Y cells following PF-04691502 treatment. PI3K (**A**), p-mTOR/total mTOR (**B**), BAX (**C**), and Bcl-2 (**D**) proteins were quantified by ELISA kits. BAX/Bcl-2 ratio is shown in (**E**). Data are indicated as mean ± SD of three independent experiments. One-Way ANOVA. *** *p* < 0.001 vs. CTR; ^##^ *p* < 0.01 vs. MPP^+^; ^###^ *p* < 0.001 vs. MPP^+^.

PF-04691502 at concentrations of 0.5 and 1 μM significantly decreased PI3K and p-mTOR/mTOR activation (Figure 6A,B), confirming that PF-04691502 potently inhibited these pathways also in this pathological context. The 0.1 μM concentration did not show any significant effect compared to the MPP^+^ group (Figure 6A,B).

Furthermore, we evaluated apoptotic markers BAX and Bcl-2 to investigate whether PF-04691502 could influence cell survival through apoptosis regulation. BAX, a pro-apoptotic protein, showed significantly lower levels in the PF-04691502-treated cells compared to the MPP^+^ group, indicating a reduction in apoptotic signaling (Figure 6C). In contrast, Bcl-2, an anti-apoptotic protein, was significantly upregulated in cells treated with higher concentrations of PF-04691502 (0.5 and 1 μM), suggesting that PF-04691502 may promote cell survival by enhancing anti-apoptotic mechanisms (Figure 6D).

Given that the BAX/Bcl-2 ratio is a widely recognized indicator of a cell’s susceptibility to apoptosis, where a higher ratio reflects a pro-apoptotic state, we evaluated this parameter to further elucidate the impact of PF-04691502 on cell survival.

From this assessment, it can be observed that MPP^+^ stimulation led to a significant increase in the BAX/Bcl-2 ratio compared to the Sham group, indicating enhanced apoptotic susceptibility under neurotoxic conditions (Figure 6E).

Notably, the treatment with PF-04691502 at concentrations of 0.5 and 1 μM significantly reduced the BAX/Bcl-2 ratio relative to the MPP^+^ group, suggesting a shift toward a more anti-apoptotic, pro-survival phenotype (Figure 6E). In contrast, the lowest concentration tested (0.1 μM) did not produce any significant change in the BAX/Bcl-2 ratio compared to with MPP^+^ alone (Figure 6E).

## 4. Discussion

PI3K/mTOR is recognized as a pivotal pathway in the regulation of immune cell activation, cytokine production, and cellular metabolism in the CNS [24]. Hence, targeting this pathway has arisen as an encouraging way to moderate neuroinflammation and potentially mitigate disease progression in multiple sclerosis (MS), AD, and PD [25].

Several PI3K and mTOR inhibitors have been explored for their anti-inflammatory effects in neurological disorders. For instance, Rapamycin (sirolimus) and its analogs, known as rapalogs, have a proven capability to suppress the activation of the microglia and reduce neurotoxic cytokine release [26,27]. PI3K inhibitors, such as Idelalisib, have been investigated for their immunomodulatory properties, though their CNS diffusion remains a challenge [28]. Dual PI3K/mTOR inhibitors, including Dactolisib and Omipalisib, offer the advantage of broader pathway inhibition, preventing compensatory feedback activation and leading to the more sustained suppression of inflammatory responses [29,30].

Thus, increasing our understanding of PI3K/mTOR’s role in neuroinflammation may open new perspectives for therapeutic interventions that have the potential to refine the management of neurodegeneration.

Therefore, this study investigated the potential of dual pathway inhibition as a strategy to mitigate neuroinflammation in neuronal and non-neuronal cells. Specifically, we evaluated the effects of PF-04691502, a dual PI3K/mTOR inhibitor, on inflammation-induced cellular stress, cytokine production, and NF-κB pathway activation in different cell lines such as SH-SY5Y, C6, BV-2, and Mo3.13. Moreover, to extend our conclusions, we also employed a PD-mimicking model using SH-SY5Y cells treated with MPP^+^, a neurotoxin that induces dopaminergic neuronal degeneration, to assess the levels of PI3K and mTOR as well as to examine apoptotic markers.

Neuroinflammation is implicated in the etiopathogenesis of numerous CNS disorders [1]. While inflammation in the CNS is typically protective in response to injurious stimuli or agents, chronic or excessive neuroinflammation can exacerbate disease progression [31]. In fact, in pathological CNS conditions, the persistent activation of glial cells contributes to the upsurge of pro-inflammatory species and ROS, resulting in neural tissue damage and functional decline [31].

Beyond neurodegenerative diseases, neuroinflammation is also implicated in other CNS disorders, such as epilepsy and depression, where persistent inflammation alters neuronal function and contributes to clinical manifestations [32]. In the last decade, growing research highlights that modulating neuroinflammatory pathways may offer a promising therapeutic approach for halting or slowing disease progression in these conditions. In this context, LPS, a bacterial endotoxin, is widely used to induce neuroinflammatory responses in experimental models [33]. LPS stimulates microglia reactivity and other immune cells in the CNS, creating a harmful environment that impairs neuronal survival [33]. This response mimics what is observed in various CNS diseases and allows researchers to study the underlying mechanisms of neuroinflammation while assessing the value of new therapeutic compounds [33].

In this regard, the results of this study demonstrated, for the first time, that PF-04691502 meaningfully counteracted LPS-induced inflammation and increased cell viability in LPS/IFNγ-damaged neuronal and glial cell lines.

The NF-κB signaling pathway is one of the main drivers in mediating neuroinflammation within the CNS [34]. Upon activation by pro-inflammatory stimuli, such as LPS and IFNγ, NF-κB undergoes phosphorylation, consequently promoting the expression of various pro-inflammatory species, including cytokines, chemokines, and enzymes [35]. These inflammatory mediators amplify the neuroinflammatory response, contributing to neuronal damage, glial activation, and synaptic dysfunction [36]. Chronic NF-κB activation is commonly associated with several neurodegenerative diseases, such as AD, PD, and MS, where the persistent inflammatory environment exacerbates disease progression [37].

In addition to its role in activating pro-inflammatory mediators, NF-κB also interacts with other signaling pathways that regulate cellular survival, apoptosis, and the immune response [35]. This makes it a central hub in the pathogenesis of neuroinflammation. Given its critical involvement in neuroinflammatory processes, targeting NF-κB activation presents a promising strategy for therapeutic intervention in CNS disorders.

In our study, treatment with PF-04691502 significantly restrained the NF-κB signaling pathway in all of the examined different cellular subtypes exposed to LPS and IFNγ stimuli. This reduction in NF-κB translocation led, therefore, to a remarkable decrease in the release of pro-inflammatory cytokines and enzymes, underscoring the potential of PF-04691502 in modulating the neuroinflammatory cascade.

After demonstrating the potential of PF-04691502 in modulating neuroinflammation via NF-κB inhibition, we further tested its effects in cultured cells, modeling PD using SH-SY5Y cells treated with MPP^+^. This step was essential for evaluating the broader neuroprotective properties of PF-04691502, particularly in a context more closely resembling the pathophysiology of PD. Given that PD is characterized by chronic neuroinflammation and dopaminergic cell loss, we hypothesized that PF-04691502 could not only attenuate inflammation but also provide neuroprotection against MPP^+^-induced toxicity. This further investigation is crucial to assess whether PF-04691502 can exert a dual effect, both reducing inflammation and promoting neuronal survival, thereby positioning it as a potential therapeutic agent for PD, and likely also for other neurodegenerative diseases.

In the present study, observations recorded for the PD cellular model revealed that PF-04691502 significantly preserved cell survival in MPP^+^-treated SH-SY5Y cells. This suggests that PF-04691502 not only modulates the neuroinflammatory response but also provides direct neuroprotection against MPP^+^-induced toxicity.

Dopaminergic markers are critical for assessing the function and integrity of dopaminergic neurons, particularly in the context of PD. TH, the rate-limiting enzyme in dopamine biosynthesis, serves as a key marker of dopaminergic neuron viability, and its reduction is commonly observed in PD models, reflecting neuronal degeneration [38]. Another PD hallmark is α-syn, a protein forming Lewy body aggregates, and its accumulation in dopaminergic neurons is associated with neuronal toxicity and dysfunction [38]. The DAT, responsible for dopamine reuptake at synapses, is also frequently measured as an indicator of dopaminergic activity [38]. Decreased DAT expression is regularly observed in PD and correlates with the loss of dopaminergic function. Together, these markers provide reliable insights into the health of dopaminergic neurons and are essential for understanding the mechanisms underlying PD and other neurodegenerative diseases [39].

In our study, dopaminergic markers like TH, α-syn, and DAT were carefully evaluated following PF-04691502 treatment in the MPP+-induced neurotoxic model.

Notably, the PF-04691502 treatment resulted in the significant preservation of TH expression, suggesting that the compound effectively mitigated dopaminergic neuron damage. This finding is consistent with the observed increase in cell viability, indicating that PF-04691502 may protect against the loss of dopaminergic function.

Additionally, PF-04691502 reduced α-syn and p-α-syn expression in the MPP+-treated SH-SY5Y cells. Such data suggest that PF-04691502 may help in alleviating the accumulation of toxic α-syn aggregates known to impair neuronal function and contribute to disease progression. Regarding the DAT levels, the PF-04691502 treatment also preserved these, pointing to the potential of the compound in maintaining dopaminergic signaling.

The PI3K/mTOR pathway, that is, the main focus of this study, has been shown to influence the neuronal response to stress, apoptosis, and inflammation [40]. By inhibiting PI3K and mTOR, PF-04691502 has the ability to modulate apoptosis, autophagy, and protein synthesis, all of which are crucial for neuronal integrity and survival.

Certainly, in neurological illness, as in the case of PD, the excessive activation of apoptotic signaling can contribute significantly to neuronal death, a central feature of disease progression [41]. Apoptosis is typically a controlled process, but in PD, the dysregulation of pro-apoptotic pathways, such as the upregulation of BAX and caspases, along with decreased levels of anti-apoptotic proteins like Bcl-2, leads to accelerated neuronal loss [42,43]. This contributes to the ongoing degeneration of dopaminergic neurons, especially within the substantia nigra, ultimately leading to the motor deficits that typify PD [44,45].

Here, the PF-04691502 treatment proved to avoid neuron death by constraining pro-apoptotic markers alongside increasing anti-apoptotic process, as can also be observed in the BAX/Bcl-2 ratio analysis. These results indicate that PF-04691502, modulating both PI3K and mTOR, may effectively promote neuronal survival by mitigating apoptosis and enhancing cellular resilience.

Consistent with our findings, other research has demonstrated the potential benefits of the dual PI3K/mTOR signaling cascade in therapeutic contexts.

Vyas et al. revealed that the combined inhibition of PI3K and mTOR using dactolisib significantly attenuated neuroinflammatory responses and seizure severity in an inflammatory model of epilepsy, highlighting the limitations of selective mTORC1 inhibition and the advantage of dual blockade in overcoming feedback reactivation mechanisms [29]. Similarly, Li et al. showed that emodin, a natural compound, conferred neuroprotection counteracting oxidative stress-induced damage in SH-SY5Y cells by inhibiting the PI3K/mTOR/GSK3β axis, further supporting the involvement of this signaling cascade in controlling cellular inflammation and survival [46].

Consistent with these observations, our findings suggest that the simultaneous modulation of the PI3K and mTOR signaling pathways can effectively counteract neuroinflammation processes, particularly in PD, emphasizing the broader relevance of targeting this pathway in various models of cellular injury.

## 5. Conclusions

Taken together, the data from this study demonstrate that PF-04691502 effectively attenuates neuroinflammatory responses by inhibiting the PI3K/mTOR signaling pathway in both neuronal and non-neuronal cell lines. The observed reduction in NF-κB activation and pro-inflammatory cytokine levels highlights its potential as a neuroprotective agent. Moreover, the preservation of dopaminergic cell viability in the MPP^+^-treated model further supports the compound’s ability to counteract both inflammation-driven and toxicity-induced neuronal damage. These results establish strong foundations for further investigation of PF-04691502, highlighting the effectiveness of this molecule for CNS disorders, also emphasizing the modulation of PI3K/mTOR signaling as a feasible healing approach.

Nevertheless, this study’s in vitro nature presents an important limitation, as it does not fully reflect the challenges of in vivo environments. Therefore, future in vivo analyses and clinical trials will be crucial to comprehensively evaluate the efficacy, safety, and translationality of PF-04691502 in diseases characterized by chronic neuroinflammation and neuronal degeneration.

## Data Availability

All data generated or analyzed during this study are included in this article.
